# Does Exercise Affect Cancer via Reverse Cholesterol Transport Process? A Hypothesis Which Needs to Be Clarified by Researchers

**DOI:** 10.34172/apb.2024.008

**Published:** 2023-08-27

**Authors:** Saleh Rahmati, Hasan Taherkhani, Aliasghar Zarezadehmehrizi, Lida Moradi

**Affiliations:** ^1^Department of Physical Education, Pardis Branch, Islamic Azad University, Pardis, Iran.; ^2^Laboratory of Integrated Physiology, Department of Health and Human Performance, University of Houston, Houston, TX, USA.; ^3^Department of Physical Education and Sports Sciences, North Tehran Branch, Islamic Azad University, Tehran, Iran.

## Dear editor,

 Although medical science has made significant advancements in cancer treatment, complementary therapies such as exercise are also essential for the treatment and improvement of cancer patients.^1,2^ Exercise can induce various beneficial effects on cancer patients through different mechanisms.^2,3^ However, the role of exercise in the reverse cholesterol transport (RCT) process in cancer patients has not been explored. The RCT process is responsible for transporting cholesterol from the blood to the liver,^4^ and many studies have investigated the effect of exercise on the elements of this process in relation to cardiovascular disease, particularly atherosclerosis.^5^ Recently, it has been discovered that RCT is also involved in cancer by regulating cell cholesterol.^6^ Cholesterol accumulation is a characteristic feature of tumor cells that affects membrane structure and function and the signaling pathways related to tumor growth.^6^ Scavenger Receptor Class B type 1 (SR-B1), ATP Binding Cassette Subfamily A and G Member 1 (ABCA1, ABCG1), and the Liver X receptors (LXRs) are key molecules involved in cholesterol efflux.^6^ The expression of ABCA1 and LXR appears to inhibit tumor growth.^6^ However, there are some contradictory findings that need further investigation.^6^ Overexpression of SR-B1 and ABCG1 can cause tumor formation and act as an oncogene.^6^ Several studies have explored the impact of physical activity on the elements of the RCT process.^5,7^ However, the effects of exercise on the RCT process in cancer conditions have not been fully investigated. The results of some studies appear to contradict exercise’s anti-cancer properties; for example, cancer can increase the expression of the ABCG1 gene, while exercise has been shown to increase tissue ABCG1 gene expression in non-cancerous subjects.^5^ It is unclear how much this gene will change in response to exercise in cancer conditions. Therefore, changes in the RCT process elements in response to exercise in cancer conditions require extensive investigation. These studies may lead to the discovery of new mechanisms associated with cancer.

## Competing Interests

 The author declares that there are no competing interests.

## Ethical Approval

 Ethics approval and consent to participate not applicable.

## Funding

 None.
